# CARE JUSTICE FOR UNPAID AND UNDERPAID CARE WORKERS

**DOI:** 10.1093/geroni/igad104.1493

**Published:** 2023-12-21

**Authors:** Nancy Hooyman

**Affiliations:** University of Washington, Seattle, Washington, United States

## Abstract

Scholarship on older women, family caregiving, feminist gerontology, community-based services and policy change intersected throughout my career. As a social worker, care of older adults has always been a matter of social justice. My focus now is on care justice for those who perform the critical work of long-term services and supports: unpaid family caregivers and underpaid home care workers. This lecture will examine the interrelationships among both types of care laborers, who often face structural constraints on their decision to care and whose work is devalued, marginalized and invisible. Inequities for care workers by race, immigrant status, and sexual orientation are rooted in systemic racism, sexism, xenophobia, and homophobia. This lecture presents the components of a care justice framework, informed by feminism, intersectionality, and care theory, of fundamental structural changes to value collectively the essential work of care, ensure meaningful choice to care, and reduce inequities in care labor.

